# Parkinson's Disease and Neurodegeneration: GABA-Collapse Hypothesis

**DOI:** 10.3389/fnins.2016.00269

**Published:** 2016-06-09

**Authors:** Janusz W. Błaszczyk

**Affiliations:** ^1^Department of Neurophysiology, Nencki Institute of Experimental Biology, Polish Academy of SciencesWarsaw, Poland; ^2^Department of Biomechanics, Academy of Physical EducationKatowice, Poland

**Keywords:** neurodegeneration, Parkinson disease, GABA modulators, hypothesis generation, models, theoretical

## Abstract

Neurodegenerative diseases constitute a heterogeneous group of age-related disorders that are characterized by a slow but irreversible deterioration of brain functions. Evidence accumulated over more than two decades has implicated calcium-related homeostatic mechanisms, giving rise to the Ca^2+^ hypothesis of brain aging and, ultimately, cell death. Gamma-aminobutyric acid (GABA) is the main inhibitory neurotransmitter within the central (CNS), peripheral and enteric nervous systems. It appears to be involved in a wide variety of physiological functions within and outside the nervous system, that are maintained through a complex interaction between GABA and calcium-dependent neurotransmission and cellular metabolic functions. Within CNS the Ca^2+^/GABA mechanism stabilizes neuronal activity both at cellular and systemic levels. Decline in the Ca^2+^/GABA control initiates several cascading processes leading to both weakened protective barriers (in particular the blood-brain barrier) and accumulations of intracellular deposits of calcium and Lewy bodies. Linking such a vital mechanism of synaptic transmission with metabolism (both at cellular and tissue level) by means of a common reciprocal Ca^2+^/GABA inhibition results in a fragile balance, which is prone to destabilization and auto-destruction. The GABA decline etiology proposed here appears to apply to all human neurodegenerative processes initiated by abnormal intracellular calcium levels. Therefore, the original description of Parkinson's disease (PD) as due to the selective damage of dopaminergic neurons in the mesencephalon should be updated into the concept of a severe multisystemic neurodegenerative disorder of the nervous system, whose clinical symptoms reflect the localization and progression of the most advanced GABA pathology. A future and more complete therapeutic approach to PD should be aimed first at slowing (or stopping) the progression of Ca^2+^/GABA functional decline.

## Current views on the etiology of Parkinson's disease

Neurodegenerative diseases constitute a heterogeneous group of disorders of the nervous system that are characterized by a slow but irreversible deterioration of brain functions. These diseases have devastating effects on patients and are often accompanied by tremendous physical and emotional burden not only for the patients but also for their families and friends. Parkinson's disease (PD) is progressive and the second most common neurodegenerative disorder (Brooks, 1998; Bergman and Deuschl, 2002). For decades, the clinical diagnosis of PD was based on a set of motor symptoms such as: rigidity, bradykinesia, akinesia, abnormal posture and resting tremor (Błaszczyk, 1998; Brooks, 1998; Bergman and Deuschl, 2002; Siderowf and Lang, 2012). The motor symptoms of PD have been linked with a loss of dopamine neurons in the *substantia nigra pars compacta* (SNPC) and a consequential reduction in the level of dopamine input in the striatum. These served as the neurophysiological hallmarks for diagnosis, although at least a 50–70% decrease in the nigrostriatal dopaminergic system occurs before the onset of clinical parkinsonism (Błaszczyk, 1998; Brooks, 1998; Bergman and Deuschl, 2002; Siderowf and Lang, 2012). Such significant brain lesion does not permit any effective medical treatment. To date, the therapy of PD is symptomatic, aimed at ameliorating motor symptoms.

Primary PD is referred to as idiopathic and understanding its causes has been an inspiration for many studies. Evidence accumulated over more than two decades has implicated calcium-related homeostatic mechanisms, giving rise to the Ca^2+^ hypothesis of brain aging and, ultimately, cell death (Disterhoft et al., 1996; Jagmag et al., 2016). Recently, the oxidative stress and calcium-induced excitotoxicity were considered important pathomechanisms leading to neural cell death in PD. Unfortunately, factors that make some neurons vulnerable to neurodegeneration while others remain resistant were not identified until now.

For biochemists, the formation of intracellular deposits of proteins and lipids that precede neuronal death seemed to be the most likely factor. The Lewy bodies, with deposits of alpha-synuclein (α-SNCA), turned out to be expressed robustly in neurodegenerating structures (Moore et al., 2005; Cookson, 2009; Beach et al., 2010). Pathological inclusions are thought to form in a small number of cells and-given enough time and, perhaps, a genetic predisposition-spread in a deterministic manner to distant brain regions (Braak et al., 2003, 2004; Goedert, 2015). Since α-SNCA deposition occurs early in PD, its immunohistochemistry has become the current gold standard in the neuropathological evaluation of PD.

The distribution of Lewy pathology in PD brains seems to be limited to specific brain regions and neuronal types, and develops in a structured, temporal pattern (Braak et al., 2004). The mechanisms underlying PD are believed to be cell-autonomous which implies that the same physiological or molecular events, such as the formation α-SNCA assemblies, occur independently in a large number of cells in an otherwise healthy brain. According to Braak's six-stage scheme (Table 1), the pathology begins (stages 1 and 2) when neurodegeneration and Lewy bodies are confined to the olfactory system, dorsal motor nucleus of the glossopharyngeal and vagal nerves, locus coeruleus, and reticular formation. Then, in stages 3 and 4, the neuropathological damage extends to the SNPC, other mesencephalic nuclei, the prosencephalon and mesoallocortical regions. During these stages, the motor symptoms develop and progressively worsen. The pathology gradually follows an ascending course, culminating in widespread synucleinopathy (stages 5 and 6), involving neocortical, prefrontal, and associative cortices. Consequently, in the advanced stages, severe motor disturbances are accompanied by cognitive and behavioral symptoms.

**Table 1:** **Main brain structures that are affected at different stages of the Parkinson's disease: symptoms and causes of their neurodegeneration**.

**PD Phase**	**Brain structures affected**	**Symptoms**	**Main cause of deficit**
Prodromal (Braak Stage 1 and 2)	Olfactory bulbs and olfactory nuclei	Hyposmia or anosmia (not responsive to antiparkinsonian drugs)	Damage of the dopaminergic neurons. Role of GABA undetermined yet.
	Dorsal nucleus of the vagus nerve	Dysautonomia, gastrointestinal disturbances, constipation	Ca^2+^/GABA
	Nucleus ambiguus	Glossopharyngeal control deficit	Ca^2+^/GABA
	Locus coeruleus and hypothalamus (orexin)	Sleep disturbance, anxiety	Progressing deficiency of the noradrenergic, serotonergic and dopaminergic systems due to GABA deficit
	Mesolimbic DA system nigrostriatal DA system	Depression, anhedonia, anxiety, impaired movement motivation, hypomimia	GABA deficiency and neurodegeneration of DA neurons, and glial-based synaptic dysfunction
Clinical (Braak Stage 3 and 4)	Striato-pallidal complex (input)	Bradykinesia, akinesia	Increased threshold of GABA medium spiny neurons
	Striato-pallidal complex (output)	Stiffness, tremor, bradykinesia, postural instability	Decreased spontaneous GABA activity (decline in GABA inhibition of competitive motor programs)
Late (Braak stage 5 and 6)	Striato-hippocampal complex	Cognitive and memory alterations	Ca^2+^/GABA induced neurodegeneration
	Thalamocortical system (prefrontal, motor, sensory, and cingular cortices)	Motivational, cognitive, sensory, and motor deficiencies	Ca^2+^/GABA-interaction collapse and generalized neurodegeneration

There are, however, several weaknesses in the Braak model. It does not explain the absence of clinical symptoms in subjects with widespread synucleinopathy or accumulation of α-SNCA in specific brain areas only. Moreover, the Lewy pathology has been identified in several neuronal populations other than the dopaminergic mesencephalic ones (Braak et al., 2004; Stefanis, 2012). Doubts can also be raised about the hypothesis of the proposed “gut to brain” spread of Lewy pathology, which claims that the pathology can initiate in the periphery, gaining access to the central nervous system through retrograde transport along projection neurons from the gastrointestinal tract (Goedert, 2015; McCann et al., 2016). It seems rather that the synucleinopathy may develop secondarily to intracellular calcium accumulation and thus does not fully explain the neurodegenerative processes.

Recently, the “triple hit” hypothesis has been advanced (Mosharov et al., 2009). This hypothesis assumes that too much calcium, plus a build-up of α-SNCA and increased dopamine within the cells, may trigger neuronal death in PD. Experimental observations confirmed that an increase in calcium concentration inside neurons when accompanied by intracellular accumulation of misfolded proteins, initiate apoptosis when a certain physiological threshold is crossed. The process of programmed cell death may be also accelerated by excitotoxicity due to excessive neurotransmitter (or medication) level (Mosharov et al., 2009).

The triple-hit model systematizes the views on neurodegeneration and on PD, although the fundamental problem of what causes the “triple hit” remains an open question. To answer this question, one has to elucidate: (i) what is the cause and mechanism of intracellular calcium accumulation, and (ii) why only certain neural structures are more prone to neurodegeneration, whilst in others, these processes are less abrupt.

## GABA and PD prodromal symptoms

Generally, patients with early PD have non-motor symptoms such as a decreased sense of smell, depression, and various gastrointestinal and other systemic features, which have been shown to predate the classical motor features of PD (Pellicano et al., 2007; Stefanis, 2012), and which are undoubtedly related to the deficit of GABA. These pre-motor signs and symptoms could be used to screen for PD before it reaches symptomatic Braak stage 3 (for details see Table 1).

Research results gathered to date, point to very interesting facts. Firstly, the cell types in the central nervous system exhibiting a propensity for developing Lewy pathology share several common features; neurons with long, thin, unmyelinated or poorly myelinated axons are particularly susceptible to develop lesions (Cookson, 2009; Hurley et al., 2013). Secondly, Parkinson's disease in its early stages extends far beyond the boundaries of the CNS, affecting the peripheral and enteric nervous systems. For this reason, a wide range of prodromal non-motor symptoms, related to various neural dysfunctions are observed in early PD. In preclinical symptoms, there is no absolute selectivity for any specific neurotransmitter group or brain area (Cookson, 2009). For instance, the pathophysiology of dysautonomia in PD includes the degeneration and dysfunction of autonomic nuclei such as the dorsal vagal nucleus, the nucleus ambiguus, and other medullary nuclei, which exert differential control on the sympathetic preganglionic neurons via descending pathways (Pellicano et al., 2007).

It is intriguing that approximately 80% of newly diagnosed PD patients have abnormal olfaction. The olfactory dysfunctions in PD seem to depend on damage of the dopaminergic neurons in the olfactory bulbs and olfactory nuclei and thus it can be a prodromal marker of the disease (Stefanis, 2012). It is intriguing that glia cell-derived neutrophic factor (GDNF) that is expressed during neuro-glial interactions (and which is controlled by the Ca^2+^/GABA mechanisms) may enhance survival and function of the dopaminergic neurons both, in the midbrain and in the olfactory system. From results of studies performed to date, it appears that GDNF may function as a chemo-attractant for GABAergic cells. GDNF is also a strong chemo-attractant for axons of dopaminergic neurons. Its ectopic application results in growth of the reinervating axons. Finally, another intriguing therapeutic aspect of the GDNF expression is the possibility of being regulated by physical activity (Ibáñez and Andressoo, 2016). Moreover, Vitamin D which is responsible for calcium metabolism is also a potent inducer of endogenous GDNF (Eserian, 2013).

During the premotor stages of PD other symptoms may play a role as prodromal markers of the disease (Pellicano et al., 2007). Depression and anxiety can be considered a non-motor sign of PD, potentially useful in the early diagnosis of PD. Depression is extremely frequent in PD, occurring in up to 45% of cases. Also panic attacks, phobias, or generalized anxiety disorder are frequently observed in PD patients. The pathophysiology of depression in PD is rather complex, being related to neurodegeneration of the noradrenergic, serotonergic and dopaminergic pathways in the brain (Pellicano et al., 2007). Recent studies indicate a possible diagnostic value of plasma GDNF levels in depression, but whether GDNF and related Ca^2+^/GABA mechanisms may play prominent role in the development and progression of PD-related depression is unclear at the moment (Ibáñez and Andressoo, 2016).

Intraneuronal ion equilibrium, including the optimal calcium level, can be fully recovered during sleep (Siegel, 2009). Therefore, sleep deficit may increase neurodegeneration. Virtually all PD patients develop sleep disruption, and there is evidence that the process usually begins early in the course of the disease. Sleep disturbance in PD has a multifactorial etiology, but pathological degeneration of central sleep regulation centers in the brainstem and thalamocortical pathways is probably the most relevant factor (Stefanis, 2012). Mechanisms of sleep are very complex, but the GABA system is involved in every its aspect (Siegel, 2009). For instance, preoptic neurons and, the nucleus reticularis, which forms a shell surrounding the thalamus, contains GABAergic neurons known from low-threshold calcium spikes (Siegel, 2009).

## GABA-calcium interaction

The most relevant fact is that all brain structures (and, in particular, those included in the Braak's model), although anatomically and physiologically diverse (e.g., using different combinations of neurotransmitters), share a common mechanism that controls their activity and metabolism. The control is just maintained through a complex interaction between gama-aminobutyric acid and calcium-dependent neurotransmission and calcium-dependent neuronal metabolism.

The Ca^2+^/GABA mechanism stabilizes neuronal activity both at the cellular and systemic levels. This close interaction, in addition to the well-documented role of Ca^2+^ in brain aging and neurodegeneration, allows one to hypothesize that a collapse of the GABA system may be decisive in the initiation and running of these processes. To validate the hypothesis one needs firstly, to investigate GABA-dependent control, and secondly, to show the symptoms and signs of its decline. In the latter case, the similarity in the symptoms of GABA deficiency compared with those of PD should be conclusive.

GABA is the main inhibitory neurotransmitter within the central, peripheral and enterinal nervous systems (Vaucher et al., 2000; Petroff, 2002; Watanabe et al., 2002). GABA appears to be involved in a wide variety of physiological functions in tissues outside the nervous system, including blood vessels, skeletal muscles, gastrointenstinal tract, pituitary, thyroid, adrenal gland, and thymus (Watanabe et al., 2002). Therefore, a deficit of GABA results in chronic dysfunctions in many systems.

Synaptic transmission is the primary mechanism in the physiology of the nervous system including signal transmission, adaptive adjustments, and memory. This mechanism utilizes the Ca^2+^/GABA interaction to improve the efficiency of the signal-to-noise ratio (Petroff, 2002; Yamakage and Namiki, 2002) and to adjust the relatively fast electrical neuronal activity to the slower biochemical and metabolic processes. Within neuronal networks, each action potential reaching presynaptic terminals opens voltage-dependent calcium channels causing an influx of Ca^2+^ into cells. The “calcium token” acts as an intracellular messenger. It controls both the trans-synaptic signal transmission and, simultaneously, when reaching the mitochondria, the calcium adjusts the cellular metabolism to activity-dependent demand (Vaucher et al., 2000; Hawkins and Davis, 2005). In this mechanism, the α-SNCA plays an important role in maintaining a supply and release of synaptic vesicles in presynaptic terminals in response to neuronal action potential (Berridge, 1998). In addition, mitochondrial metabolic functions depend also on the α-SNCA. Unfortunately, an excessive intracellular concentration of calcium in combination with extravagant SNCA accumulation leads to apoptosis and neurodegeneration (Mosharov et al., 2009).

## Deficit of GABA and neurodegeneration

To protect the neurons, the GABA system must precisely control the calcium influx directly via GABAergic receptors and, indirectly, via astrocytes and glial networks (Yamakage and Namiki, 2002; Allaman et al., 2011). Activation of the classical presynaptic receptors GABA_A_ and GABA_B_ results in a hyperpolarization of neurons and plays a critical role in the long-term inhibition of synaptic transmission (Watanabe et al., 2002; Yamakage and Namiki, 2002). During the hyperpolarizing phase, the voltage-gated calcium channels are blocked, thus protecting neurons from Ca^2+^ toxicity and gives them time to remove the surplus calcium ions (Mosharov et al., 2009; Hurley et al., 2013). Removal of the divalent calcium ions from the mitochondria and cytoplasm requires, however, significant amounts of energy (and time), and therefore, calcium overloaded neurons have high energy requirements (Surmeier and Schumacker, 2013). Hence, much evidence suggests a major role of mitochondrial dysfunction in the pathogenesis of PD (Moore et al., 2005). Long-lasting intracellular calcium load results in mitochondrial oxidative stress that can exacerbate neurodegeneration (Surmeier and Schumacker, 2013). Consequently, the mitochondrial production of adenosine tri-phosphate (ATP) drops rapidly and a toxic calcium excess cannot be removed from the cell. This cascade of pathological events is controlled by GABA activity that may precisely dose the amount of calcium entering the cell (Walker and Semyanov, 2007; Mosharov et al., 2009; Glass et al., 2010). A decline in this calcium buffering capacity is just responsible for neuronal loss in the *substantia nigra* in PD (Hurley et al., 2013; Surmeier and Schumacker, 2013).

**Figure 1:**
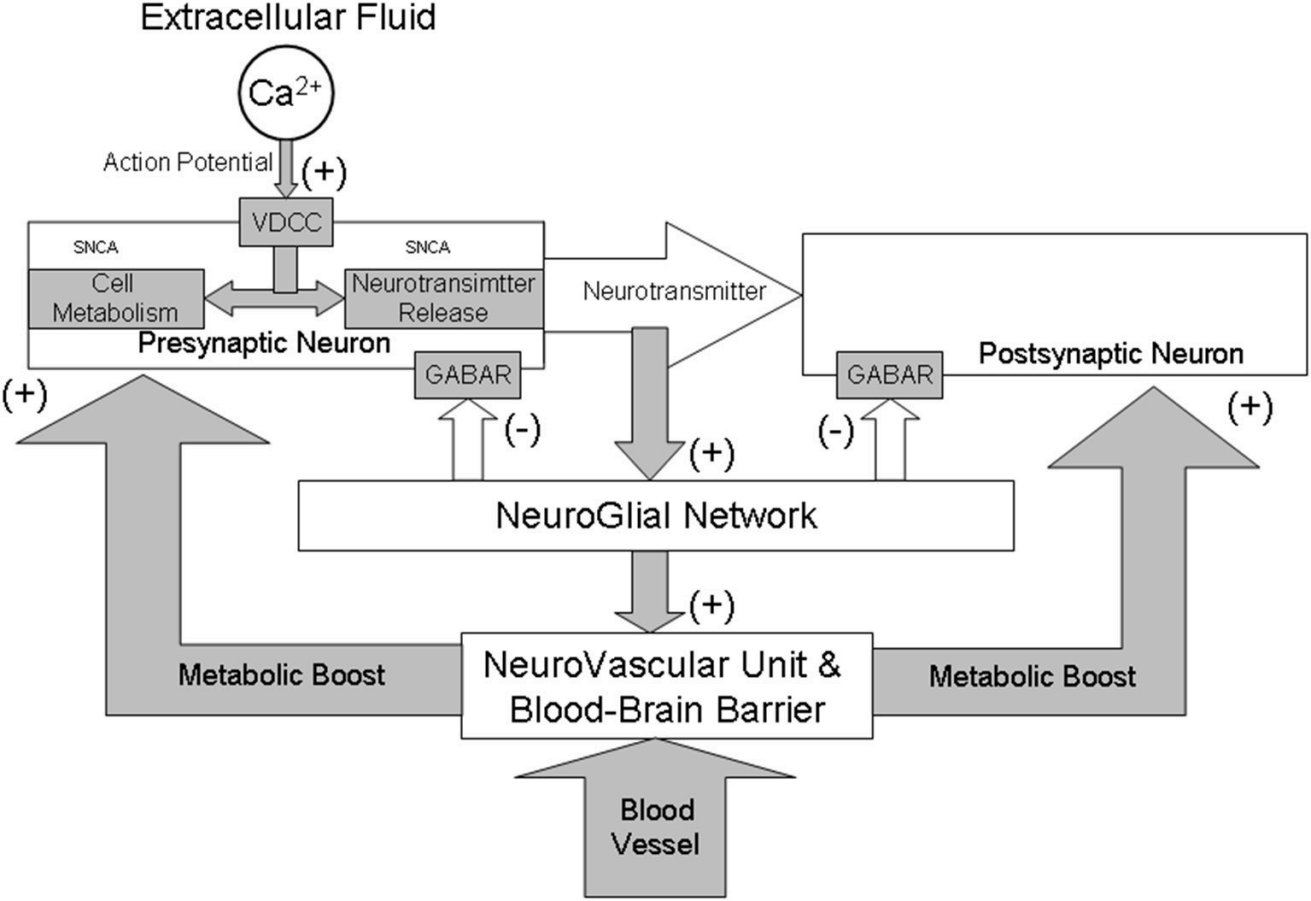
**A schematic illustration of neuro-glial interactions during synaptic transmission from a functional perspective of GABAergic system**. All brain structures, although anatomically and physiologically diverse, share a common mechanism that controls their activity and metabolism. The control is maintained through a complex interaction between gamma-aminobutyric acid (GABA) and calcium (Ca^2+^) dependent neurotransmission and cellular metabolic functions. Activation of both classical GABA_A_ and GABA_B_ receptors (GABAR) results in a hyperpolarization of neurons and play a critical role in long-term inhibition of synaptic transmission. The α-SNCA plays an important role in these interactions by maintaining a supply/release of synaptic vesicles and the mitochondrial metabolic function in a calcium-dependent manner. Generally, Ca^2+^/GABA mechanism stabilizes neuronal activity both at cellular and systemic levels. The collapse of this mechanism may be decisive in the initiation and running of brain aging and neurodegeneration due to “triple hit” (too much calcium plus a build-up of α-SNCA and excitotoxicity). Collapse of the GABA inhibition results in vasodilation which may change permeability the blood-brain barrier and may initiate inflammation of blood vessels and brain tissue thus intensifying neurodegenerative processes in PD.

There is also another potent mechanism that controls calcium-mediated processes within the CNS, and which, when fails may initiate of neurodegeneration. The release of neurotransmitters within neuronal networks activates neighboring astrocytes that in turn, via GABA, inhibit the further influx of calcium ions into the presynaptic neurons (negative feedback in Figure 1). Astro-neuronal networks are self-regulating systems (Rial et al., 2016). Excessive neuronal activity is firstly tuned by increased GABA inhibition and, if not successful, the density of GABA synaptical receptors and the number of calcium channels are reduced (Petroff, 2002; Moore et al., 2005; Surmeier and Schumacker, 2013). When these mechanisms fail, the excitability of the neuronal network is adjusted in the process of excitotoxicity that eliminates neurons with excessive activity. When a neurotransmitter concentration around the synaptic cleft cannot be decreased or reaches a higher level (e.g., due to neuronal overexcitation) the neuron kills itself by a process of apoptosis.

As a general role, GABA interneurons act as homeostatic regulators of synaptic inhibition within principal cell networks. GABA is released into the extracellular space and is then transported into neurons and glial cells (Hawkins and Davis, 2005; Allaman et al., 2011). If the ambient GABA concentration decreases, the neurons become more excitable (Walker and Semyanov, 2007). Generally, the efficiency of GABA mediated mechanisms depends on its concentration within the nervous system. The concentration depends on the amount of GABA that is synthesized and released, as well as on the activity of enzymes and cofactors involved in its processing. In addition to GABA release, there is also GABA uptake, the efficiency of which varies from brain region to brain region and even within specific regions (Richerson and Wu, 2003). The astrocytes, besides regulating neurotransmitter levels, also support the metabolism of neurons (Allaman et al., 2011; Rial et al., 2016). For instance, they release large amounts of lactate in the extracellular space that can diffuse through the astrocytic network and rescue neuronal activity during glucose deprivation.

A balance between excitation and inhibition ensures the faultless functioning of neuronal and astro-neuronal networks (Figure 1). To maintain the balanced activity of the brain the glutamate-GABA-glutamate recycling process is used. Astrocytes uptake glutamate and synthetize GABA in a calcium-dependent manner (Walker and Semyanov, 2007). In this process, the L-glutamic acid decarboxylase (GAD) and vitamin B6 as a cofactor are used (Petroff, 2002). GAD exists in two isoforms: GAD65 and GAD67 that synthesize GABA at different locations in the neurons, at different developmental times, and for functionally different purposes (Pinal and Tobin, 1998). Only GAD65 synthesizes GABA for neurotransmission. After its Ca-inhibitory action, GABA is recycled through the tricarboxylic acid cycle back to glutamate. Recently, it became evident that defects of these astrocytic functions and/or alterations of astrocyte–neuron interaction, results in neuronal damage (Allaman et al., 2011). This finding offers a promissing therapeutic strategy in PD. New therapy consisting of insertion of the glutamic acid decarboxylase gene into the subthalamic nucleus that results in partial but significant relief of symptoms of Parkinson's disease (LeWitt et al., 2011).

## Ca^2+^/GABA and PD motor symptoms

The only output of the nervous system is the motor system, whether in cognition or action (Grillner et al., 2005). It is also reasonable to hypothesize that PD motor symptoms are mainly due to basal ganglia deficiency (Błaszczyk, 1998; Brooks, 1998; Bergman and Deuschl, 2002; Błaszczyk et al., 2007; Cenci, 2007). The basal ganglia play an essential role in adaptive motor control by allowing relevant motor programs to be executed while inhibiting potentially competing movements (Grillner et al., 2005; Cenci, 2007). In this control, the pallidal output keeps the different sensori-motor and motivational centers (including *thalamus, ventral tegmental area*, and *substantia nigra*) and their target neurons under tonic (90 Hz) GABA inhibition (Grillner et al., 2005). This unique spontaneously active GABA output may be particularly vulnerable to aging and neurodegeneration. A decline in the tonic GABA inhibitory activity of the basal ganglia results in increasing co-activation of different competitive motor programs (Cenci, 2007). This, in turn, causes co-activation of a variety of muscle groups, including co-contractions of agonist and antagonist muscles and progressive stiffness, which leads to progressive changes in posture and a rigid gait (Błaszczyk et al., 2007; Błaszczyk and Orawiec, 2011). GABA compounds that cross the blood-brain barrier or increase GABA activity alleviate muscle stiffness caused by a lack of GABAergic tone. Also the use of GABA-producing transplants for recovery of function in the rat Parkinson model introduces a novel concept of therapeutic intervention in Parkinson's disease (Winkler et al., 1999).

Movement initiation and rapid transition between motor programs at the level of basal ganglia are critically modulated by a dopaminergic projection from the SNPC to the striatum. The importance of this projection is illustrated by the dopamine-dependent motor symptoms of PD (Cenci, 2007). Reduced dopamine innervation of the striatum, as in PD, results in hypokinesia and difficulty in initiating different motor programs. Significantly, some of the cardinal PD motor symptoms: postural instability, tremor, and freezing phenomenon do not always improve with levodopa, and, in fact, can be made worse.

An interesting feature of midbrain dopamine neurons and the other brainstem nuclei that degenerate in Parkinson's disease is that they are autonomously active (pacemaker activity), with prominent transmembrane calcium currents that generate regular, slow, broad action potentials (2–4 Hz) in the absence of synaptic input (Surmeier and Schumacker, 2013). Neurons in the *substantia nigra pars compacta* and the *subthalamic nucleus* (STN) preferentially use CaV1.3 channels for pacemaking which makes them more susceptible to calcium mediated excitotoxicity and neurodegeneration. Thus, GABA inhibition of Cav1.3 channel activity may be neuroprotective for the remaining *substantia nigra pars compacta* neurons in patients with Parkinson's disease (Hurley et al., 2013). Also fast-spiking neurons of the STN, that constitute the “central pacemaker of the basal ganglia,” became targets for transplants of protective GABA releasing cells (Winkler et al., 1999; LeWitt et al., 2011). In both cases, the tight interaction of neuro-glial units seems to be the main, if not the only, protective mechanism against damage. Recently, the protective effects of glial cell-derived neurotrophic factor (GDNF) for midbrain dopaminergic neurons have been also reported (Eserian, 2013; Ibáñez and Andressoo, 2016). Interestingly, the neuroprotective effects were observed only when the GDNF was delivered into the gabaergic striatum, but not directly to the SNPC dopaminergic neurons (Ibáñez and Andressoo, 2016). This finding strengthens the proposed GABA- collapse hypothesis.

The input GABA neurons of the basal ganglia have a high threshold for activation and are essentially silent (Grillner et al., 2005). An increasing threshold of striatal input due to GABA deficiency would be manifested in bradykinesia and hypokinesia. In this case, GABA deficiency at the striatal input to the basal ganglia would require increased dopamine input. GABA striatal spiny neurons forming an input system to the basal ganglia are only activated during motor activity and they do not seem to degenerate (Cenci, 2007).

Parkinsonian akinesia has been attributed to an imbalance between movement-suppressing and movement-promoting pathways within the basal ganglia (Cenci, 2007). In fact, all the motor symptoms of PD may be explained solely by malfunctions in the spinal and supra-segmental inhibitory networks that utilize GABA (Figure 1).

## GABA and blood-brain barrier

Brain activity depends also on the balance between neuronal activity and its metabolic demands. Neurodegeneration and PD pathology may depend on brain metabolism (Figure 1). Given the dynamic nature of brain activity and the considerable metabolic needs of bioelectrically active nervous tissue, the microcirculation of the brain must be highly responsive to the tissue it supplies. Anatomical evidence confirmed a direct innervation of the microvascular endothelium by GABAergic neurons (Vaucher et al., 2000). GABA activity allows the maintenance and optimization of metabolic, neurotrophic and energetic supply of the brain by controlling neurovascular units (Hawkins and Davis, 2005). This neuronal function depends also on the Ca^2+^/GABA mechanisms. A normal level of GABA helps maintain tightness and selectivity of the blood-brain barrier.

While the exact cause of chronic neurodegeneration of PD is not known, increasing evidence suggests that chronic inflammation is the fundamental process mediating the progressive nature of the neurodegeneration characteristic of PD (Glass et al., 2010). Induced by GABA deficit, inflammation may amplify the pathology. Collapse of GABA inhibition results in the relaxation of smooth muscle within the brain vessels. Long-term, this may change the permeability of the blood brain barrier (BBB) and thus may initiate inflammation of blood vessels and brain tissue thereby intensifying neurodegenerative processes. Damaged or poorly functioning BBB is more susceptible to inflammatory responses involving microglia and astrocytes that may contribute to PD progression (Glass et al., 2010). Many environmental factors may influence inflammatory responses that contribute to neurodegenerative pathologies, including traumatic injury, systemic infection, diet and prolonged occupational exposure to metals (Migliore and Coppedè, 2009; Kwakye et al., 2015). Anatomical evidence has been found for direct control of the neurovascular units and/or associated astrocytic processes by excitatory neurotransmitters. The Ca^2+^/GABA system plays a vital function here and its deficiency can lead to the disruption of the BBB (Hawkins and Davis, 2005).

In conclusion, physiological control mechanisms within the nervous system, and particularly the combined control of the neuron's activity and its metabolism, suggests that deficiency in only one of these mechanisms may be responsible for the progressive decline in brain function and neurodegeneration. In this context, the GABA decline etiology appears to apply to all human neurodegenerative processes initiated by abnormal intracellular calcium levels. Linking such a vital mechanism of synaptic transmission (and neuronal bioelectrical activity) with metabolism (both at cellular and tissue level) by means of a common reciprocal Ca^2+^/GABA inhibition results in a fragile balance which is prone to destabilization and auto-destruction. The process(es) is initiated in cells that are the weakest (less active or overactive) within different brain regions. Then, depending on genetic predisposition as well as hormonal and environmental factors, the process can accelerate with rapidly progressing degradation of the nervous system.

Decline in the Ca^2+^/GABA control initiates several cascading processes leading to both weakened protective barriers (in particular, the blood-brain barrier) and accumulations of intracellular deposits of calcium and the Lewy bodies. A future and more complete therapeutic approach to PD should be aimed firstly at slowing (or stopping) the progression of Ca^2+^/GABA functional decline and thus slowing the aging/degeneration processes within the nervous system. In this context, to implement screening for early PD, it will be important to develop a more precise understanding of the time-course of the emergence of prodromal features of PD and the timing of the onset of GABA threshold impairment that leads to a progressive and irreversible neurodegradation and calcium-dependent apoptosis. In conclusion, the original description of PD as due to the selective damage of dopaminergic neurons in the mesencephalon should be up-dated into the concept of a severe multisystemic neurodegenerative disorder of the brain, whose clinical symptoms reflect the localization and progression of the most advanced GABA pathology.

## Author contributions

The author confirms being the sole contributor of this work and approved it for publication.

### Conflict of interest statement

The author declares that the research was conducted in the absence of any commercial or financial relationships that could be construed as a potential conflict of interest.
